# Evidence for Multiple Rhythmic Skills

**DOI:** 10.1371/journal.pone.0136645

**Published:** 2015-09-16

**Authors:** Adam Tierney, Nina Kraus

**Affiliations:** 1 Auditory Neuroscience Laboratory, Northwestern University, Evanston, Illinois, United States of America; 2 Department of Communication Sciences, Northwestern University, Evanston, Illinois, United States of America; 3 Institute for Neuroscience, Northwestern University, Evanston, Illinois, United States of America; 4 Department of Neurobiology and Physiology, Northwestern University, Evanston, Illinois, United States of America; 5 Department of Otolaryngology, Northwestern University, Evanston, Illinois, United States of America; Max Planck Institute for Human Cognitive and Brain Sciences, GERMANY

## Abstract

Rhythms, or patterns in time, play a vital role in both speech and music. Proficiency in a number of rhythm skills has been linked to language ability, suggesting that certain rhythmic processes in music and language rely on overlapping resources. However, a lack of understanding about how rhythm skills relate to each other has impeded progress in understanding how language relies on rhythm processing. In particular, it is unknown whether all rhythm skills are linked together, forming a single broad rhythmic competence, or whether there are multiple dissociable rhythm skills. We hypothesized that beat tapping and rhythm memory/sequencing form two separate clusters of rhythm skills. This hypothesis was tested with a battery of two beat tapping and two rhythm memory tests. Here we show that tapping to a metronome and the ability to adjust to a changing tempo while tapping to a metronome are related skills. The ability to remember rhythms and to drum along to repeating rhythmic sequences are also related. However, we found no relationship between beat tapping skills and rhythm memory skills. Thus, beat tapping and rhythm memory are dissociable rhythmic aptitudes. This discovery may inform future research disambiguating how distinct rhythm competencies track with specific language functions.

## Introduction

Rhythm is an integral part of our daily lives. Across cultures, music uses durations of sounds and silences to form repetitive rhythmic patterns [1]. Musicians, dancers, and listeners use rhythm to perform, synchronize to, and remember music. Similarly, speech contains rhythmic patterns on a number of time scales, from the slowing that occurs at the ends of phrases and sentences [2–5] to the lengthened durations that indicate stress in English [6]. This rhythmic information can help listeners pick out the component sounds of speech by facilitating detection of word boundaries [7–11] and stress patterns [7].

Rhythmic skill, therefore, may not only determine one’s success as a musician or dancer, but may also have wider consequences for language. Performance on a variety of rhythm skills has been linked to linguistic abilities. For example, rhythm discrimination tracks with verbal working memory [12–13] and grammatical skill [14]. Hausen et al. [15] reported that the ability to detect whether a sound has shifted off-beat correlates with word stress perception. Performance on several different rhythm tests tracks with reading ability, including discriminating rhythms [13,16–17], remembering and repeating back rhythms [18–20], synchronizing to a metronome [20–24], tempo reproduction [20,25], tapping to the beat of music [26], and perception of musical meter [27].

There is growing evidence, therefore, that language skills rely on rhythmic abilities that are not domain-specific and can therefore be assessed using non-linguistic stimuli. Consequently, rhythmic deficits may underlie delayed or disrupted language development in some children, which, if true, could lead to targeted assessment and rehabilitation. However, in order to successfully untangle the links between rhythm and language, it is necessary to understand rhythmic skill itself, and at present there is little agreement as to what are the fundamental rhythmic abilities involved in perceiving and producing speech or music. One possibility is that there is a single rhythmic competency underlying all of the rhythm skills mentioned above. If this were true, then perhaps a single rhythmic task might suffice for measuring a participant’s rhythmic skill, an assumption that underlies the use of tests of rhythmic aptitude which include only a single rhythm measure [12,28–29]. Supporting this idea, multiple studies have shown correlations between tests of a variety of rhythm skills. For example, Fujii and Schlaug [30] found weak-to-moderate correlations between performance on tests of tapping to the beat of music, metrical discrimination, and tracking tempo changes. Fitch and Rosenfeld [31] found that the ability to tap to the beat of a rhythm correlated with rhythm reproduction and rhythm discrimination. Pecenka and Keller [32] found that the ability to predict progressive tempo changes during beat tapping predicted accuracy when synchronizing with a partner. Finally, Keele et al. [33] found that motor timing variability correlated with perceptual timing accuracy, suggesting that rhythm perception and production form a single skill set.

An alternate hypothesis is that there are multiple rhythmic ‘intelligences’. Several lines of evidence converge to suggest that rhythm is best characterized as a multi-dimensional skill set. First, the brain networks underlying perception of sub- versus supra-second intervals are dissociable, with sub-second intervals linked to the cerebellum and frontal operculum and supra-second intervals linked to premotor and prefrontal cortex [34–35]. This suggests that rhythm skills may be dissociable based on whether they rely on perception of patterns in time at sub-second versus supra-second rates. Second, perception of metrically simple rhythms is linked to increased activation in subcortical and cortical motor structures such as the basal ganglia and supplementary motor area [36], suggesting that metrical rhythm perception may be dissociable from non-metrical rhythm perception. Finally, evidence from case-studies suggests that beat tapping and rhythm sequence perception are dissociable: problems with beat-tapping can coincide with preserved rhythm discrimination [37–40], and vice versa [41]. To date, however, evidence for this dissociation between beat tapping and rhythm sequence perception has come from isolated case studies rather than by examining performance across rhythm tests in the general population.

Here we set out to test the hypothesis that there are multiple dissociable rhythm skills by assessing several skills in individual participants, then examining relationships between performance on these tests. We predicted that performance on tests of metronome tapping and tempo adaptation would correlate, as beat-tapping relies upon the ability to use auditory feedback to correct for transient fluctuations in the timing relationship between motor output and auditory input [42–43]. We also predicted that performance on tests which require remembering and reproducing a temporal sequence would correlate, as both of these tests require subjects to hear a pattern of durations, remember that pattern, and produce a motor sequence that will reproduce the pattern. However, given the evidence from case studies that rhythmic skills may not be a unitary phenomenon, we hypothesized that beat-tapping and rhythm memory would not be linked. To test these hypotheses we assessed participants on a battery of four rhythm tests that align themselves in two dimensions: 1) beat-tapping: tapping to a metronome and rapid adaptation to a change in metronome tempo, and 2) memory/sequencing: drumming along to a repeating temporal sequence and reproducing a temporal sequence from memory.

## Methods

### Participants

67 participants were recruited (33 female) with a mean age of 17.99 years (standard deviation 1.02). All had normal hearing (pure tone thresholds < 20 dB for octaves between 250 and 8000 Hz) and no history of a neurological or language disorder. Informed written consent was obtained from participants over the age of 18. For participants under the age of 18 informed written assent was obtained, and written consent was obtained from the participants’ legal guardians. All procedures were approved by the Institutional Review Board at Northwestern University.

### Rhythm testing

All four rhythm tests used the same system for stimulus presentation, collection of drumming data, and marking of stimulus and drum onset times. Stimuli were stored and presented on an iPod Nano (Apple) and participants were asked to drum with one hand on a conga drum. A vibration-sensitive drum trigger pressed against the underside of the head of the drum picked up the timing of the participant’s drum hits. A copy of the audio signal presented to participants via headphones and the output of the drum trigger were combined as two channels of a stereo input to a computer running the audio recording program Audacity. Thus, the participant’s drumming and the stimulus to which the participant was listening were simultaneously recorded to a single sound file.

Continuous stimulus and drum data were converted to a list of onsets by a custom-written Matlab program. This program marked drum onsets at any point in the stimulus or drum signal which satisfied two preconditions: 1) the amplitude at that point exceeded a preset amplitude threshold, and 2) the point was preceded by a length of time equal to or greater than a preset time threshold during which no point exceeded the amplitude threshold. This “refractory period” ensures that multiple adjacent high-amplitude time points resulting from a single drum hit are not marked as several drum hits. Because the exact manner in which the drum is hit can vary among participants, the marked onsets were visually compared to the raw continuous data and any errors were corrected by manually adjusting the time and amplitude thresholds. Amplitude thresholds were set to be as small as possible without resulting in false alarms, i.e. background noise being marked as drum onsets. This ensured that individual differences in the amplitude of drum hits did not affect the reliability with which drum hit onset times could be marked. These stimulus and drum onsets were then subjected to further analyses for each rhythm test, as described below.

### Beat-tapping: Metronome

This test assessed participants’ ability to drum consistently along to an isochronously presented stimulus. Six trials were presented. Each trial consisted of a snare drum stimulus (duration 99 ms, acquired at freesound.org) repeated 40 times with a constant inter-onset-interval (IOI). In two of these trials the IOI was 667 ms (1.5 Hz presentation rate), in two the IOI was 500 ms (2 Hz), and in two the IOI was 333 ms (3 Hz). These trials were always presented in this order. Participants were asked to drum along to the metronome such that their drum hits occurred at the exact same time as the sounds they were hearing. To give the participant ample time to begin synchronizing to the beat, only the last twenty beats of each trial were analyzed. The consistency of each participant’s drumming was assessed by calculating the standard deviation of the IOI of the drum hits within each trial and dividing by the IOI of the stimulus for that trial, then averaging across all six trials.

### Beat-tapping: Tempo adaptation

This test assessed how quickly participants could adapt to a change in tempo. Fifty-five trials were presented. Each trial consisted of a conga drum sound (duration 150 ms, acquired at freesound.org) repeated isochronously between 6 and 10 times with a 500 ms IOI, followed by five more presentations. (This same conga drum sound was used in all of the remaining rhythm tests described below.) In five of the trials these last five sounds continued to be presented with a 500 ms IOI, i.e. there was no shift in tempo. In twenty-five of the trials the last five sounds were presented with a shorter IOI (faster tempo), with five trials each using IOIs of 450, 460, 470, 480, and 490 ms. In the remaining twenty-five trials the last five sounds were presented with a longer IOI (slower tempo), with five trials each using IOIs of 510, 520, 530, 540, and 550 ms. Participants’ ability to switch to the new tempo was assessed by calculating, for each of the last two intervals the participant produced in each of the fifty trials containing a tempo shift, the absolute value of the difference between the target IOI and the produced IOI. These two values were then averaged to form a tempo adaptation score for that trial. Performance across all fifty shifted trials was then averaged to compute an overall tempo adaptation score.

### Memory/sequencing: Drumming along to rhythmic sequences

This test assessed participants’ ability to rapidly perceive and drum along to a temporal sequence. Four trials were presented. In each trial, a 3.2-second four-measure sequence taken from Povel and Essens [44] was repeated ten times, for a total of forty measures. Each four-measure sequence consisted of the conga sound presented nine times. Every sequence used the same set of IOIs: five 200 ms, two 400 ms, one 600 ms, and one 800 ms. The sequences differed in the order in which these IOIs were presented, which gave rise to different temporal patterns. Two of the trials contained sequences taken from the set of strongly metrical sequences listed in Povel and Essens [44], while two of the trials were weakly metrical sequences which contained more rests in strongly metrical positions (greater syncopation). Participants were asked to listen to the sequences and then, once they had a good idea of what the sequences were, drum along, aligning their movements exactly with what they heard.

Both the stimulus and drumming data were converted to a sequence of hits and rests. First, it was determined whether each 200 ms time interval in the stimulus contained a drum hit or silence. The drumming data was then similarly converted to a sequence of hits and rests: if a drum hit took place between 100 ms before and 100 ms after the onset of a given 200 ms stimulus interval a hit was added to the drum sequence, otherwise a rest was assumed. The test was then scored by comparing the sequence of drum hits and rests to the sequence of stimulus hits and rests. For example, if the stimulus sequence were [0 1 1 0] and the drumming sequence were [1 1 1 0], where a zero indicates a rest and a one indicates a hit, the participant’s score on this small section of the test would be 75%. Performance was calculated across the second through tenth repetitions of each rhythm and averaged across all four trials.

### Memory/sequencing: Remembering temporal sequences

This test assessed participants’ ability to remember and repeat back a temporal sequence. Thirty trials were presented. During each trial a 3.2-second four-measure sequence from Povel and Essens [44] was repeated three times, followed by a pause equal in length to the duration of the sequence. During this pause the participant attempted to repeat the sequence exactly when it would have occurred had it repeated a fourth time. The first fifteen trials consisted of strongly metrical sequences, while the second fifteen trials consisted of weakly metrical sequences. As described above for the drumming along to temporal sequences task, performance on this task was analyzed by converting the stimulus and drumming data to sequences of hits and rests, and then compared to assess performance. Drum hits and rests were marked by searching for drum onsets in a 200-ms window centered around the time points when each stimulus interval would have begun had the stimulus repeated a fourth time. Performance was averaged across all thirty trials.

### Verbal memory

Our goal was to determine whether beat-tapping and rhythm memory/sequencing form dissociable rhythm skills. However, this analysis relies upon the assumption that these tasks test rhythm skills, rather than (or in addition to) more general cognitive abilities. In particular it is important to establish that performance on the rhythm memory/sequencing tasks cannot entirely be accounted for by general memory skill. To test this participants were given the Auditory Working Memory (AWM) and Digits Reversed (DR) subtests from the Woodcock-Johnson III Test of Cognitive Abilities [45].

### Statistical analysis

One participant was an outlier on metronome tapping variability (> standard deviations from the mean); this participant was removed from all analyses. For no other measures were there outliers greater than three standard deviations from the mean. A second participant was removed from analysis because during the metronome test the average difference between the mean tempo they produced and the metronome tempo was 38.7 ms. All other participants were within 5 ms of the target IOI on average. The Jacque-Bera test for normality revealed that the paced variability and drumming along to rhythmic sequences measures were not normally distributed (p < 0.05). As a result, we log-transformed the paced variability and rau-transformed the drumming to rhythmic sequences data, after which these measures were normally distributed (p > 0.05).

**Fig 1:**
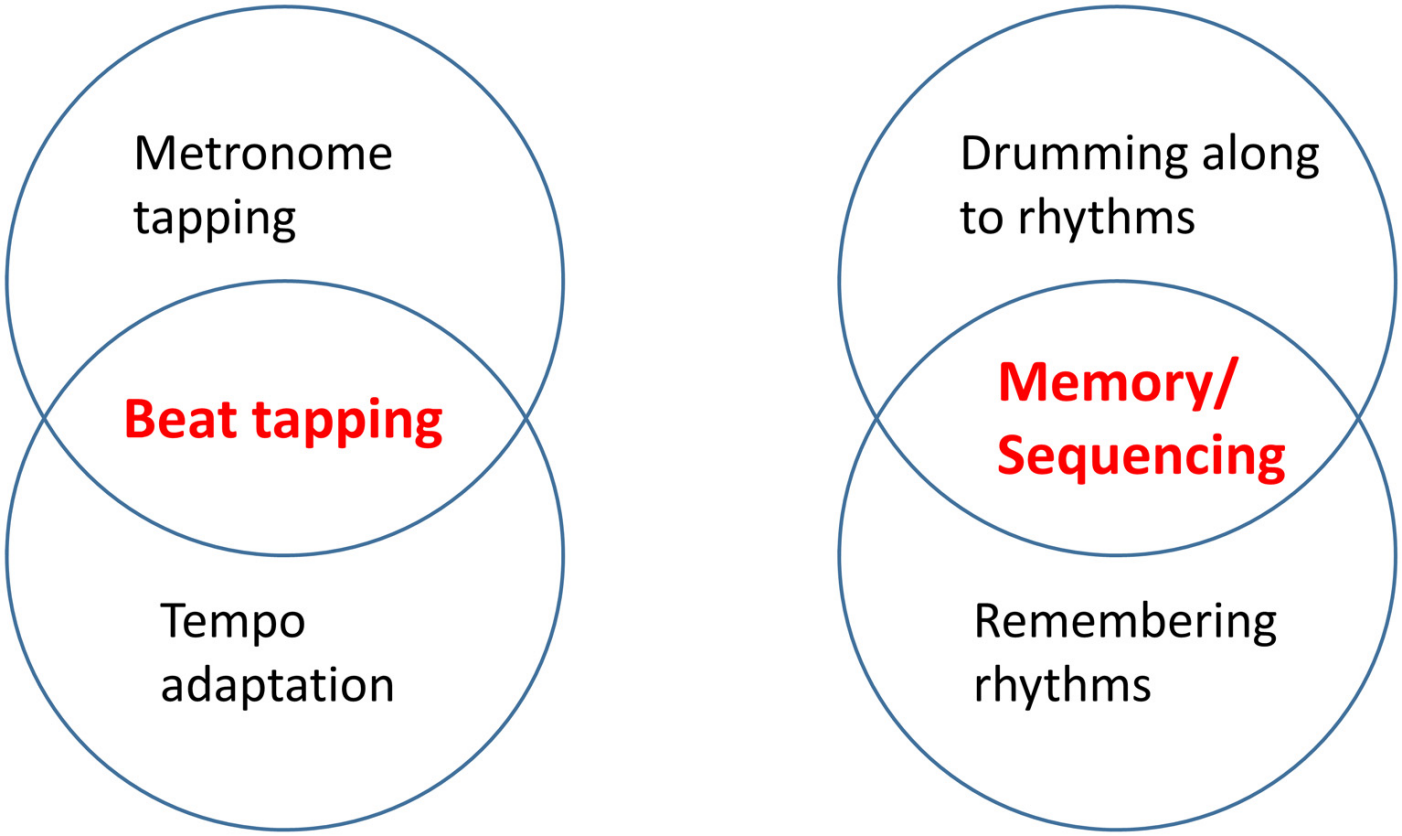
Schematic of hypothesized relationships between performance on Beat tapping and Memory/Sequencing tests.

Performance across all four measures of rhythm ability was correlated using Person’s correlations. If all four measures broadly assess a single rhythmic competence, all four measures should correlate with one another. Conversely, we hypothesized that metronome tapping and tempo adaptation would be linked, due to a shared reliance on fine temporal acuity, and that drumming along to and remembering temporal sequences would be linked, as these tests require subjects to remember temporal patterns and produce a motor sequence that will reproduce them. However, we predicted that metronome tapping variability and temporal adaptation (beat-tapping set) would not relate to drumming along to and remembering temporal sequences (memory/sequencing set). See Fig 1 for a schematic of predicted relationships between performance on these rhythm tests. As a further test for the existence of multiple rhythm skills a generalized least squares factor analysis with varimax rotation was used to uncover latent variables reflecting shared variance across rhythm measures.

## Results

**Fig 2:**
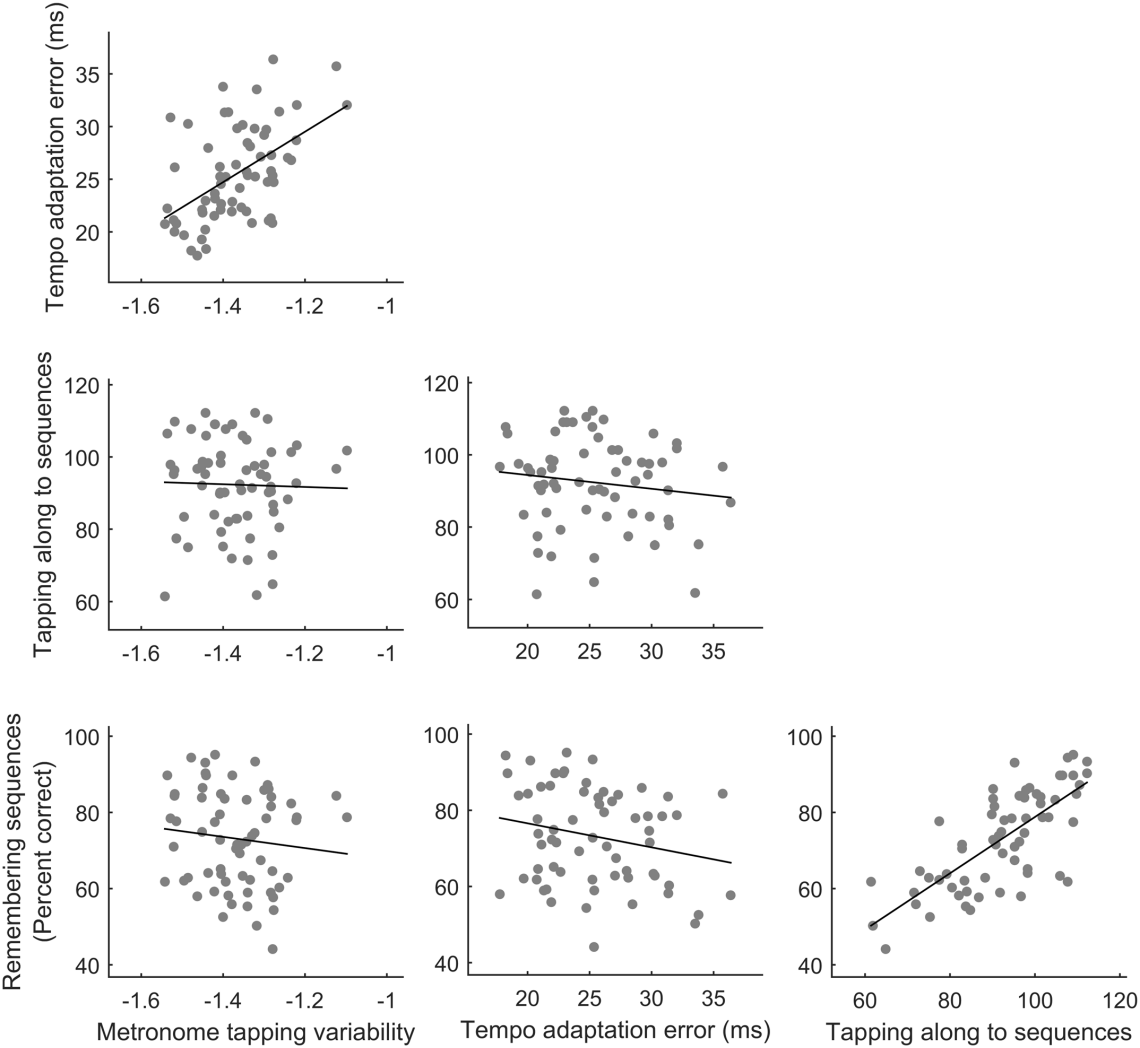
Scatterplots displaying the relationship between performance on rhythm tests. Tempo adaptation error correlated with metronome tapping variability (r = 0.51, p < 0.001), and remembering sequences correlated with drumming along to sequences (r = 0.73, p < 0.001). No other correlations were significant (p > 0.05). To ensure normality of data paced variability data has been log-transformed and tapping along to sequences data has been rau-transformed.

Fig 2 shows scatterplots comparing performance across all four rhythm tests, while r-values and p-values for each correlation can be found in Table 1. Participants who tapped more consistently to a metronome were also better able to shift to a new metronome tempo (r = 0.51, p < 0.001). Moreover, participants who were highly accurate at drumming along to a temporal sequence also were more accurate when reproducing temporal sequences from memory (r = 0.73, p < 0.001). However, neither beat tapping measure (metronome tapping variability nor tempo adaptation) related to either memory measure (drumming along to or remembering temporal sequences (all p > 0.05)). Furthermore, factor analysis revealed that performance across the rhythm tests was best captured by two factors, which together accounted for 81% of the variance across the data set. The first factor consisted of performance on the drumming to sequences and remembering sequences tests. The second factor consisted of performance on the metronome tapping and tempo adaptation tests. See Table 2 for factor loadings across all four rhythm tests for each of the two factors.

**Table 1:** r-values and p-values for Pearson’s correlations comparing performance on all four rhythm tests. Significant relationships are indicated via boldface.

r-value, p-value	Metronome tapping	Tempo adaptation	Drumming to sequences	Remembering sequences
Metronome tapping				
Tempo adaptation	**0.51, 1.2x10** ^**-5**^			
Drumming to sequences	-0.03, 0.82	-0.14, 0.28		
Remembering sequences	-0.11, 0.37	-0.22, 0.07	**0.73, 4.8x10** ^**-12**^	

**Table 2:** Factor loadings from generalized least squares factor analysis. Boldface indicates loadings of greater than 0.3.

	Factor 1	Factor 2
Metronome tapping	0.049	**0.882**
Tempo adaptation	-0.165	**0.857**
Drumming to sequences	**0.928**	0.010
Remembering sequences	**0.917**	-0.126

**Fig 3:**
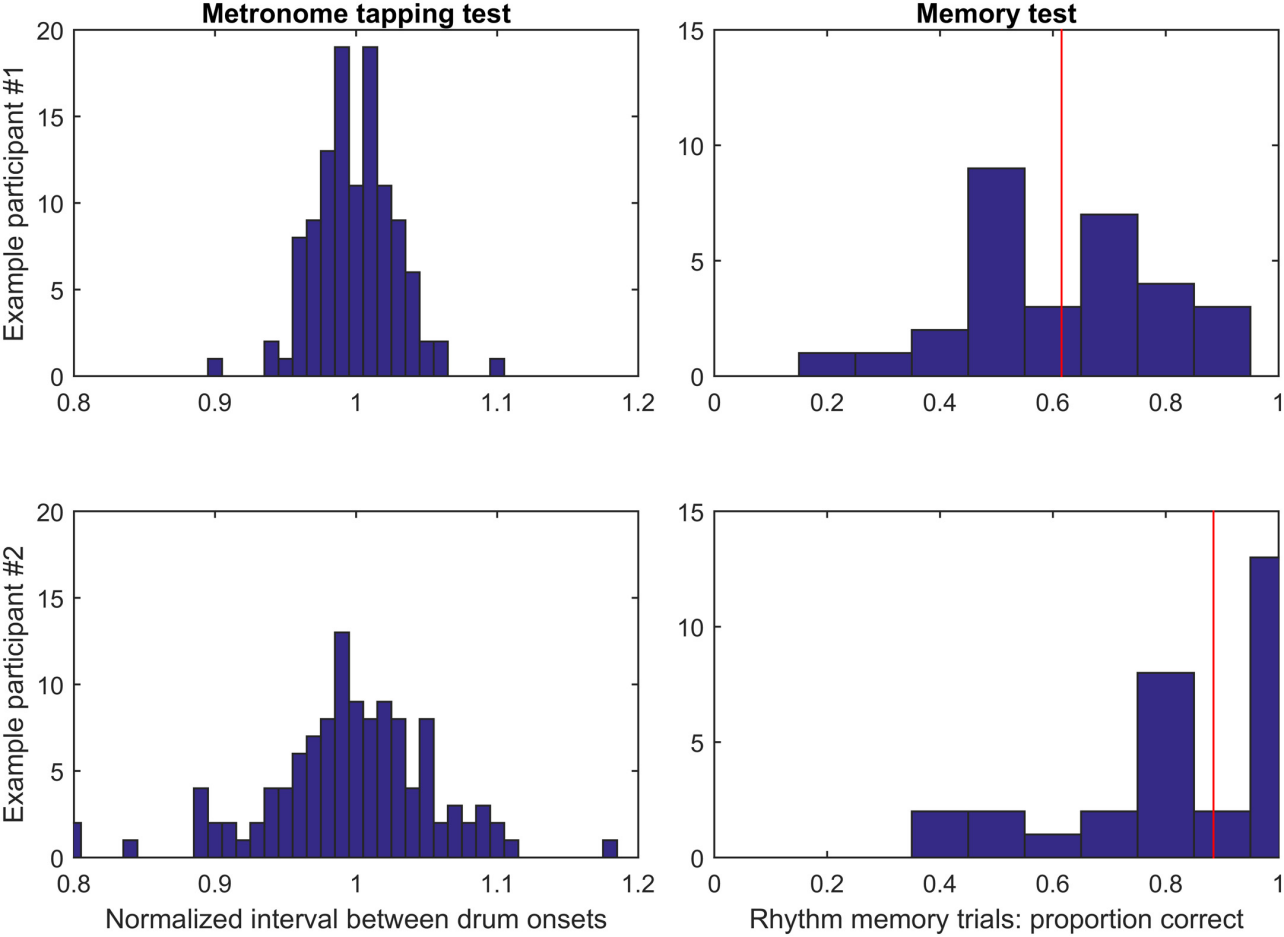
Performance on metronome tapping and sequence memory tests for two example participants. The left-hand column displays a histogram of intervals between drum onsets normalized by the target inter-onset-interval of each condition (for example, an interval of 550 ms in the 500 ms condition would be converted to 1.1). The right-hand column displays a histogram of scores on each of the 30 rhythm memory trials; the vertical red line indicates median performance. Example participant #1 (top) could tap consistently to a metronome but performed poorly on the memory test, while example participant #2 (bottom) tapped variably to a metronome but was able to remember the majority of the rhythms.

To illustrate the extent to which performance on beat tapping versus memory/sequencing tests was uncoupled, Fig 3 displays performance on beat tapping and memory/sequencing tests in individual subjects. Example subject #1 (Fig 3, top) tapped with little variability, producing a normalized metronome tapping variability of only 0.029, compared to the median across all participants of 0.043. Their ability to rapidly shift to a new metronome tempo was similarly impressive, with an average error of only 20.7 ms, putting them in the top 13% of participants tested in this study. However, their performance on the drumming to sequences test (62.3% correct) and the sequence memory test (61.8% correct) was quite low. Example subject #2 (Fig 3, bottom), on the other hand, performed more poorly on the beat tapping and tempo adaptation tests than every participant but one, producing a normalized metronome tapping variability of 0.079 and an average tempo adaptation error of 36.4 ms. However, their performance on both the drumming to sequences test (92.6%) and the sequence memory test (84.4%) was better than average.

To determine whether the rhythm sequencing/memory tasks assess truly rhythmic skills or whether they simply reflect general memory capacity we assessed the relationship between performance on these rhythm tasks and verbal memory. Correlations between the rhythm memory tests and the verbal memory tests were weaker than correlations between the two rhythm memory/sequencing tests: drumming to sequences correlated with backwards digit span at r = 0.25 (p < 0.05) and with verbal auditory working memory at r = 0.26 (p < 0.05), while remembering sequences correlated with backwards digit span at r = 0.38, p < 0.01 and with verbal auditory working memory at r = 0.35, p < 0.01). To determine whether verbal memory and rhythm memory are dissociable we ran two hierarchical regressions. In the first, we entered auditory working memory and backwards digit span on the first step and found that they predicted 17% of the variance in memory for rhythmic sequences (p < 0.01). However, adding drumming to sequences performance on the second step improved model fit by 41% (p < 0.001). For the second regression, we entered drumming to sequences and memory for sequences performance on the first step, and found that they predicted 15% of the variance in backwards digit span (p < 0.01). However, adding auditory working memory on the second step improved model fit by 25% (p < 0.001). Thus, verbal memory and rhythmic memory are overlapping but dissociable skills.

## Discussion

Here we find that the ability to consistently synchronize and the ability to quickly adapt to a changing metronome tempo while synchronizing are linked (beat tapping measures). We also find that participants who are better able to remember rhythms and reproduce them are also better able to drum along to those rhythms (memory/sequencing measures). However, there was no relationship between either test of beat tapping and either test of rhythm memory/sequence processing. These results support the theory that there are multiple dissociable rhythm skills, but do not support the existence of a single overarching rhythmic competence or “rhythm IQ”.

If beat tapping and rhythm memory/sequencing are separable skills they may also rely on dissociable neural foundations. Neuroimaging studies have shown links between beat tapping and the cerebellum, basal ganglia, and primary motor cortex [46–49]. While rhythm memory/sequence perception has been linked to these same areas, it has also been associated with the premotor cortex and the supplementary motor area [50–54]. One difference between the neural structures on which these two sets of tasks rely, therefore, seems to be that the rhythm memory/sequence set relies upon motor planning regions in addition to the subcortical and primary cortical motor regions on which beat tapping depends. Furthermore, beat tapping may place more stringent demands on cerebellar processing than does rhythm sequence perception, as children with cerebellar lesions have been shown to be impaired on a beat tapping task but not a rhythm discrimination task that requires remembering rhythmic sequences [39]. Alternately, rhythm memory and beat tapping may rely on different subregions of the cerebellum. Beat tapping may also place greater demands on the stable representation of microsecond timing of sounds in the subcortical auditory system [55].

A crucial difference between beat tapping and rhythm memory/sequence processing that may drive the dissociation between the two sets of tasks is that the rhythm memory/sequence set requires integration of acoustic information across a greater length of time than does beat tapping. To track even a relatively short rhythm spanning a few measures requires retaining and integrating temporal information over several seconds, while the auditory-motor error-correction processes that comprise the beat tapping set operate over time spans of tens of milliseconds [56–59]. Reviews of the time perception literature [60–63] have indicated a dissociation between time perception at the millisecond scale, which is associated with the cerebellum [34,63–70], and time perception at the supra-second time scale, associated with the basal ganglia and cortical regions [63,65,70]. Integration of temporal information over longer durations may require motor planning processes [71]. Motor planning areas (premotor and supplementary motor areas) have strong internal connections that may make self-sustaining activity possible, enabling integration of information across longer durations, while cerebellar circuity lacks such internal excitatory connections [72].

Developing an informed taxonomy of rhythm skills could have important consequences for the study of links between rhythm aptitude and language proficiency. Previous studies of links between rhythm and language skills have used a wide variety of rhythm tasks, including rhythm discrimination [12–14.16–17], detection of off-beat sounds [15], remembering and repeating back rhythms [18–20], synchronizing to a metronome [20–24], tempo reproduction [20,25], tapping to the beat of music [26], and perception of musical meter [27]. Learning how these rhythm skills relate to one another could lead to insights regarding the relationship between rhythm and language. For example, different rhythm aptitudes may be linked to different language functions. Beat-tapping may not be linked to verbal memory because a simple continuous beat is easy to extract and because strictly periodic beats are not present in spoken language, but the temporal precision of auditory perception required by auditory-motor error-correction processes may also be vital for discrimination of speech sounds and development of phonological awareness [73]. Conversely, remembering rhythmic sequences may be more strongly linked to verbal memory, given the non-periodic rhythmic patterns which are found in speech [74].

It should be noted that all of our rhythm tasks require both perception and production. How are perception and production integrated and dissociated? A recent paper introducing a battery of tests of beat processing [30] reported surprisingly low correlations between perceptual and production metrics produced by each test. Moreover, unattended musical rhythms can affect perceptual processing, even when modalities of the rhythms and perceptual task differ [75–76]. Rhythm perception and production skill may, therefore, be dissociable. Given that our tasks here included both perceptual and production elements, future work should determine whether rhythm perception on fast and slow time scales can be dissociated in the absence of a production component.

Rhythm memory and verbal memory were dissociable skills, with the drumming to sequences task accounting for an additional 40% of the variance in the sequence memory task over the variance accounted for by both verbal memory tests. Nevertheless, rhythm memory and verbal memory were related: the rhythm memory/sequencing tests predicted 15% of the variance in backwards digit span scores, supporing the link between auditory short-term memory span and rhythm reproduction reported by Grahn and Schuit [77]. These results suggest that verbal memory depends to a limited extent on perception of and memory for temporal patterns. Efficient tracking of temporal patterns in speech such as the lengthened durations that mark phrase boundaries and stressed syllables may facilitate detection of these elements, freeing up cognitive resources which can then be devoted to rehearsal and manipulation of the material to be remembered.

Categorizing rhythm abilities into clusters that reflect the neural foundations on which they draw could have important clinical consequences. While there is some preliminary evidence that rhythm-based training can improve language skills [78–80], these studies have used wide-ranging training regimens which trained many different rhythm skills. Understanding how language relies on rhythm within the framework of the two rhythm competencies discovered here (beat tapping and memory/sequencing) could lead to targeted interventions for the subset of children whose language problems stem from these specific rhythmic deficits.
